# renz: An R package for the analysis of enzyme kinetic data

**DOI:** 10.1186/s12859-022-04729-4

**Published:** 2022-05-16

**Authors:** Juan Carlos Aledo

**Affiliations:** Departamento de Biología Molecular Y Bioquímica, Universidad de Málaga, 29071 Málaga, Spain

**Keywords:** Computer program, Progress curve, Integrated rate equation, Michaelis–Menten, K_M_, V_max_

## Abstract

**Background:**

Complex enzymatic models are required for analyzing kinetic data derived under conditions that may not satisfy the assumptions associated with Michaelis–Menten kinetics. To analyze these data, several software packages have been developed. However, the complexity introduced by these programs is often dispensable when analyzing data conforming to the canonical Michaelis–Menten model. In these cases, the sophisticated routines of these packages become inefficient and unnecessarily intricated for the intended purpose, reason for which most users resort to general-purpose graphing programs. However, this approach, in addition of being time-consuming, is prone to human error, and can lead to misleading estimates of kinetic parameters, particularly when unweighted regression analyses of transformed kinetic data are performed.

**Results:**

To fill the existing gap between highly specialized and general-purpose software, we have developed an easy-to-use R package, renz, designed for accurate and efficient estimation of enzyme kinetic parameters. The package provides different methods that can be clustered into four categories, depending on whether they are based on data fitting to a single progress curve (evolution of substrate concentration over time) or, alternatively, based on the dependency of initial rates on substrate concentration (differential rate equation). A second criterion to be considered is whether the experimental data need to be manipulated to obtain linear functions or, alternatively, data are directly fitted using non-linear regression analysis. The current program is a cross-platform, free and open-source software that can be obtained from the CRAN repository. The package is accompanied by five vignettes, which are intended to guide users to choose the appropriate method in each case, as well as providing the basic theoretical foundations of each method. These vignettes use real experimental data to illustrate the use of the package utilities.

**Conclusions:**

renz is a rigorous and yet easy-to-use software devoted to the analysis of kinetic data. This application has been designed to meet the needs of users who are not practicing enzymologists, but who need to accurately estimate the kinetic parameters of enzymes. The current software saves time and minimizes the risk of making mistakes or introducing biases due to uncorrected error propagation effects.

## Background

Enzymes not only make metabolic reactions kinetically possible, but they also modulate the rates of these reactions in a coordinated way, thus responding duly to the changing needs of the cell, reason for which enzymes have been described as “agents of life” [1]. Knowledge of enzyme kinetics is critical for understanding biological systems, as illustrated in recent publications [2–4], but also has an industrial interest [5]. A canonical approach to enzyme kinetics makes use of the Michaelis–Menten equation [6, 7], which describes the dependence of enzyme-catalyzed reaction rates on the concentration of substrate. An enzyme is said to follow a Michaelis–Menten reaction mechanism if the initial rate of the catalyzed reaction obeys the equation:

1 $$v = V_{\max } \frac{\left[ S \right]}{{K_{M} + \left[ S \right]}}$$

More complex enzymatic models are required for analyzing kinetic data derived under conditions that may not satisfy the assumptions associated with Michaelis–Menten kinetics [8]. To analyze these data and assist in the selection of the best enzymatic model, a number of software packages, such as DynaFit [9], KinTek [10], and ENZO [11], have been developed. However, the additional complexity introduced by these programs is often dispensable when analyzing data conforming to the canonical model summarized by the Michaelis–Menten equation. In these cases, the sophisticated routines of these packages become inefficient and unnecessarily intricate for the intended purpose. For these reasons, most users often resort to general-purpose graphing programs such as GraphPad Prism or Microsoft Excel. However, this approach, in addition of being time-consuming and prone to human error, can lead to misleading estimates of kinetic parameters, particularly when unweighted regression analyses of transformed kinetic data are performed [12]. A recently developed tool designed to overcome part of the above pointed drawbacks is ICEKAT [13], a browser-based tool for semi-automated initial rate calculations. The user can upload series of kinetic traces and download the resulting table of initial rates. Although this resource represents a valuable tool, the offered menu of methods for estimating the kinetic parameters (K_M_ and V_max_) is limited. Although, in general, the performance regarding availability and speed of web-based tools is beyond the control of the user, in the case of ICEKAT the source code is freely available, which opens the possibility to run the software locally if wished.

Herein, we present renz, an R package specifically designed for the analysis of Michaelis–Menten kinetic data. We have taken advantage of the fact that R is a free, popular and powerful environment for statistical computing and graphics. Furthermore, renz package compiles and runs on a wide variety of UNIX platforms, Windows and MacOS as a stand-alone program (Table 1), which allows fast and efficient performance of the utilities implemented in this package. Finally, the package is accompanied, in addition to the standard documentation, by diverse vignettes that illustrate the use of the software and guide the user on the most suitable method, discussing the pros and cons of the different alternative analyses available.

**Table 1:** Comparison of different software programs for fitting enzyme kinetic data

Software	Free open source	Cross-platform	Stand-alone
renz	✓	✓	✓
ICEKAT	✓	✓	–
DynaFit	✓	–	✓
KinTek	–	–	✓
ENZO	✓	✓	–

## Implementation

### What renz is and how to start using it

**Fig. 1:**
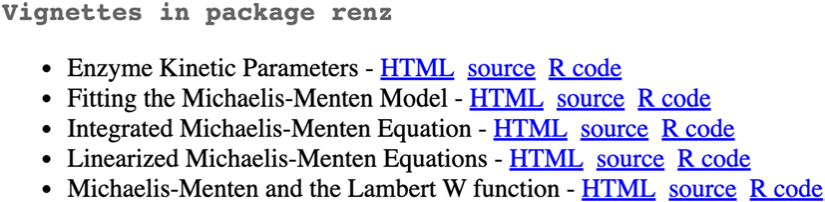
The accompanying vignettes can be visualized and navigated through the user browser. When typing into the R console: *browseVignettes(“renz”)*, the user’s browser opens a new tab offering a menu like the one shown in this figure

renz is an R package containing utilities for the analysis of enzyme kinetic data. It is currently distributed as a platform independent source code under the GPL version 3 license. The user, before attempting to install renz, must have a relatively recent version of R (≥ 4.0.0) installed and running on their system. Detailed instructions for obtaining and installing R on various platforms (Mac, Linux, and PC) can be found on the R home page (https://www.r-project.org). We also encourage to install RStudio (https://www.rstudio.com), a useful integrated development environment for R. Afterwards, open R and type into the console: *install.packages(“renz”)*. At this point the user is ready to start exploring the utilities provided by renz. It should be noted that, if the user has not previously installed the *knitr* and *rmarkdown* packages, some warnings may be thrown by the console during the installation of *renz*. In that case, the user can either ignore them (the performance of *renz* will not be affected) or, alternatively, opt to install *knitr* and *rmarkdown*, required for properly formatting the documentation related to the accompanying vignettes. To guide the exploration of *renz*, we have made five vignettes, through which we can navigate with our browser. For instance, when you type into the R console *browseVignettes(“renz”)*, your browser should show an appearance like the one shown in Fig. 1. The first of these vignettes, entitled “Enzyme Kinetic Parameters” presents an overview of the different methods implemented into the current package to estimate K_M_ and V_max_ of a Michaelis–Menten enzyme. The remaining vignettes present, in a brief way, the theoretical principles of the different methods available to determine the kinetic parameters and illustrate with examples the use of the package functions that carry out the corresponding analyses.

## Results and discussion

### An overview of methods to estimate the kinetic parameters

**Fig. 2:**
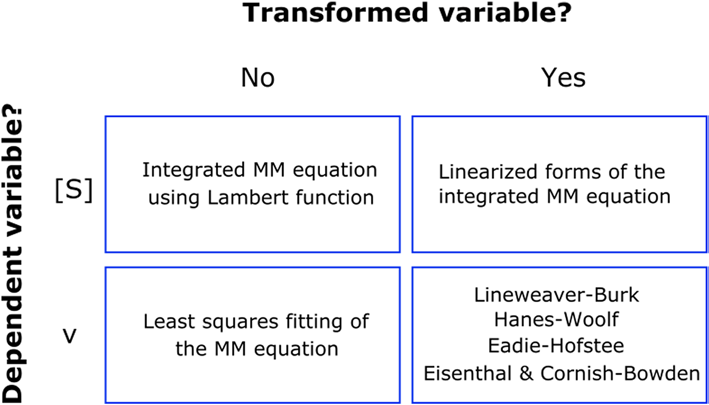
Classification of available methods to estimate the kinetic parameters of a Michaelis–Menten enzyme. The clustering of methods attends to two binary criteria: (i) whether or not it is necessary to transform the original variable, and (ii) what the dependent variable determined experimentally is, substrate concentration or initial rate. MM stands for Michaelis–Menten

The methods for estimating K_M_ and V_max_ can be grouped into four categories (Fig. 2), according to two criteria: (i) which is the considered dependent variable, and (ii) whether it is necessary to transform the original variables.

Regarding the first of these criteria, when the method uses data where the substrate concentration is the dependent variable that evolves over time (independent variable), then a single progress curve is enough to determine the kinetic parameters. That is, the data consist of a set of (t, [S]) points. Those researchers interested in designing an experiment to collect and analyze this type of data, could benefit from the advice provided in [14]. Alternatively, when the method uses the initial velocity (dependent variable) as a function of the substrate concentration (independent variable now), several progress curves will be required. In this case, attention should be paid to the experimental design [15], as well as to the procedure to compute the initial rate from the progress curve [16]. In any event, since each initial rate is estimated from the early linear portion of each progress curve, we will need as many progress curves as ([S], v) points we want to use in order to determine K_M_ and V_max_. Therefore, in absence of any other consideration, the first group of methods, where the entire progress curve is fitted to an integrated rate equation is preferable because they imply less work on the bench, and data are used more efficiently than in the initial rate assay [17, 18]. Furthermore, they can be more accurate because they avoid underestimation of initial rates, particularly under conditions where [S]/K_M_ is low [19].

With respect to the second criteria, those methods that do not transform the original variables are preferable. The reason is that we are dealing with experimental data, that is, the dependent variable is always affected by an indeterminate amount of error. When this variable is transformed, errors will propagate in a non-homogeneous way. In other words, error propagation strongly depends on the algebraic manipulations introduced to transform the original variables. This error distortion, when ignored, can lead to severe bias in the estimation of K_M_ and V_max_. In the vignette “Linearized Michaelis–Menten Equations” we illustrate, using real experimental data, how easily one can be misled by this bias related to uncorrected error propagation.

**Fig. 3:**
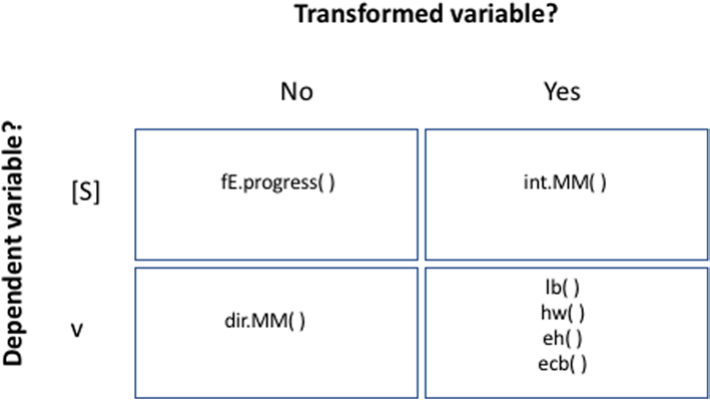
Summary of functions from renz available to determine the kinetic parameters of a Michaelis–Menten enzyme. The functions are clustered following the same criteria used in Fig. 2. Help (standard documentation) for the use of each function can be found within the package, typing into the R console the question mark followed, without spaces, by the name of the function. For instance: *?lb*. More detailed documentation and examples can be found in the specific vignettes, as shown in Fig. 1

The package renz offers functions that allow to estimate the enzyme kinetic parameters using any of the four approaches we have introduced above (Fig. 3). To check the format of the required input data and the format of the returned output, the user can type into the R console the name of the function preceded by a question mark, for instance: *?dir.MM.* A more detailed documentation for each function, including examples, can be found in the provided vignettes (Fig. 1).

### Case-study: use of initial rates to estimate the kinetic parameters of the *β-*galatosidase enzyme

In this section we want to emphasize the need to rigorously analyze experimental data to avoid misleading estimates of kinetic parameters. Given the specific aim of this section, many relevant information that must be reported when publishing an enzyme function article has been omitted herein. In this regard, a complete and useful guide can be found at [20, 21]. We used *β*-galatosidase (EC. 3.2.1.23) as an enzyme model, which catalyzes the hydrolysis of o-nitrophenyl-*β*-d-galactopyranoside (ONPG) to galactose and o-nitrophenol. The influence of the substrate concentration on the initial rate of this reaction was determined by octuplicate at 10 different ONPG concentrations. Full data are included in renz package and can be obtained typing into the R console: ONPG. However, in this section we will focus our attention on a single dataset (Table 2) that illustrates well how easily wrong conclusions can be drawn when the analysis is not adequate.

**Table 2:** Initial rate *versus* substrate concentration

[ONPG] (mM)	0.05	0.10	0.25	0.50	1.00	2.50	5.00	8.00	20.00	30.00
v (µM min^−1^)	3.0	6.0	17.0	31.0	48.0	101.0	121.0	139.0	152.0	181.0

The table shows one of the eight replicates included into the package renz, which can be consulted typing on the R console: ONPG

**Fig. 4:**
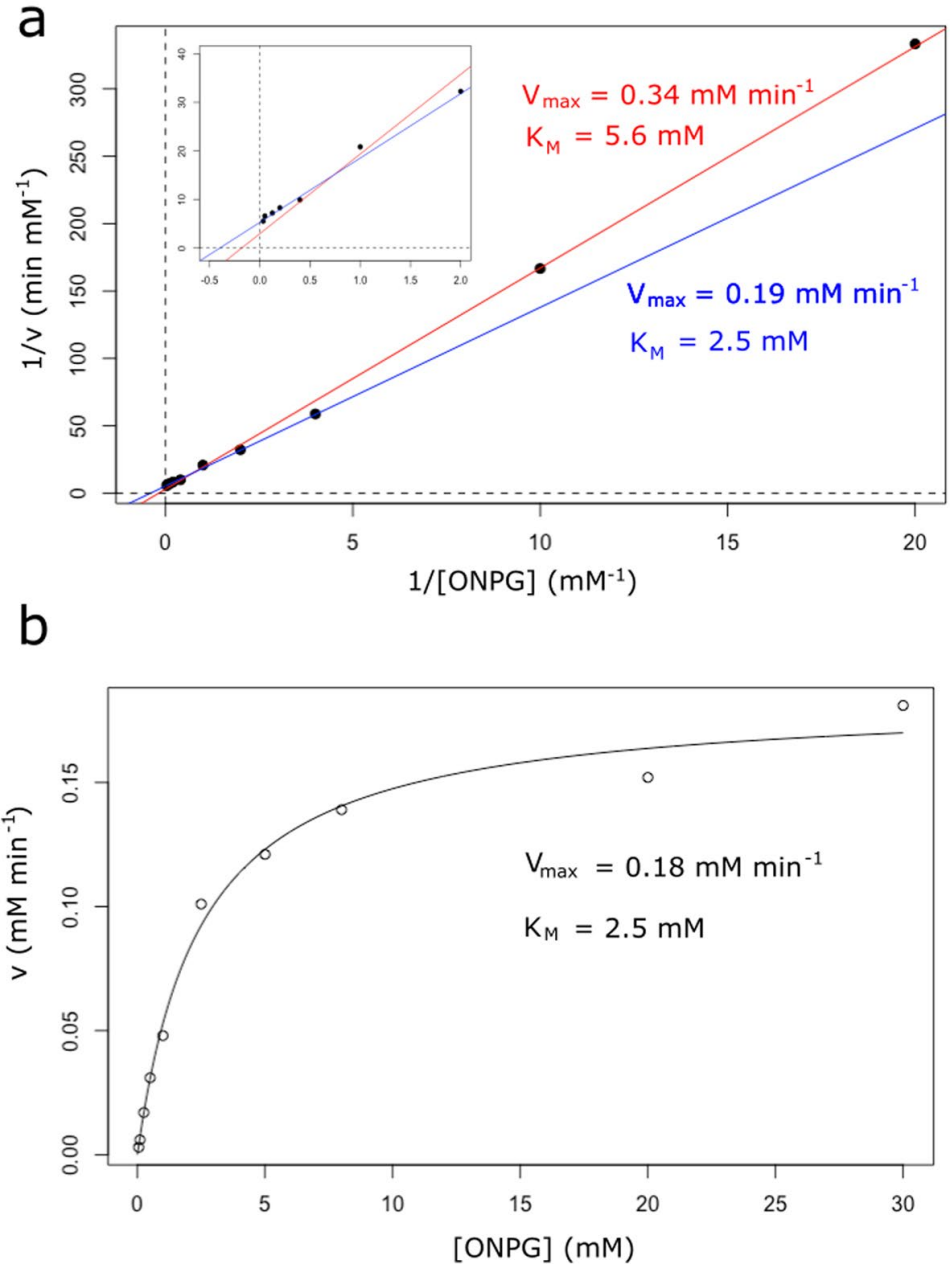
Weighted linear regression and non-linear regression avoid the risk of obtaining biased kinetic parameters. **a** Using the data given in Table 2, the double-reciprocal plots obtained without weights (red line) and with weights (blue line) are shown. The inset focus on the points with higher weights in the weighted regression analysis. **b** The function *dir.MM()* from the renz package also allows for direct non-linear regression analysis. The curve and parameters obtained using this function are shown in this plot

If we take double-reciprocal from data shown in Table 2 and fit them to a line equation, as many general-purpose software users would do, the estimated parameters are: K_M_ = 5.6 mM and V_max_ = 0.34 mM min^−1^. However, if we implement weighted linear regression when performing our double-reciprocal analysis (i.e., *lb(data, weighting* = *TRUE)*), the obtained parameters are now: K_M_ = 2.5 mM and V_max_ = 0.19 mM min^−1^ (Fig. 4a), which represent values more in line with those obtained when data are directly fitted to the Michaelis–Menten equation using non-linear least square techniques (i.e., *dir.MM(data)*): K_M_ = 2.5 mM and V_max_ = 0.18 mM min^−1^ (Fig. 4b).

### Case-study: predicting the evolution of substrate concentration over time

For most enzyme-catalyzed hydrolysis reactions, the kinetic parameters can be estimated using the integrated Michaelis–Menten equation.

2 $$\frac{1}{t}\ln \frac{{S_{o} }}{\left[ S \right]} = - \frac{1}{{K_{M} }}\frac{{\left( {S_{o} - \left[ S \right]} \right)}}{t} + \frac{{V_{\max} }}{{K_{M} }}$$

With *S*_*o*_ representing the initial substrate concentration, while [*S*] is the substrate concentration at time *t*. K_M_ and V_max_ can be obtained after transforming the ($t, \left[ S \right]$) data into $\left( {\frac{{\left( {S_{o} - \left[ S \right]} \right)}}{t},\frac{1}{t}\ln \frac{{S_{o} }}{\left[ S \right]}} \right)$ and fitting them to Eq. (2). Of course, this task can be conveniently performed all in one go, using a single command from the renz package: *int.MM(data)*. Although the use of this function saves time and minimizes the risk of introducing human error, users can still turn to a general-purpose program to perform linear fit. In either case, as we have discussed above, the algebraic manipulations that accompany the linearization of equations, in which the original variables (affected by experimental error) are not linearly related, entail some bias. Therefore, to obtain robust estimate of the kinetic parameters, it is much more convenient to fit ($t, \left[ S \right]$) data to an explicit solution for the integrated Michaelis–Menten equation known as the Schnell-Mendoza equation [22].

**Fig. 5:**
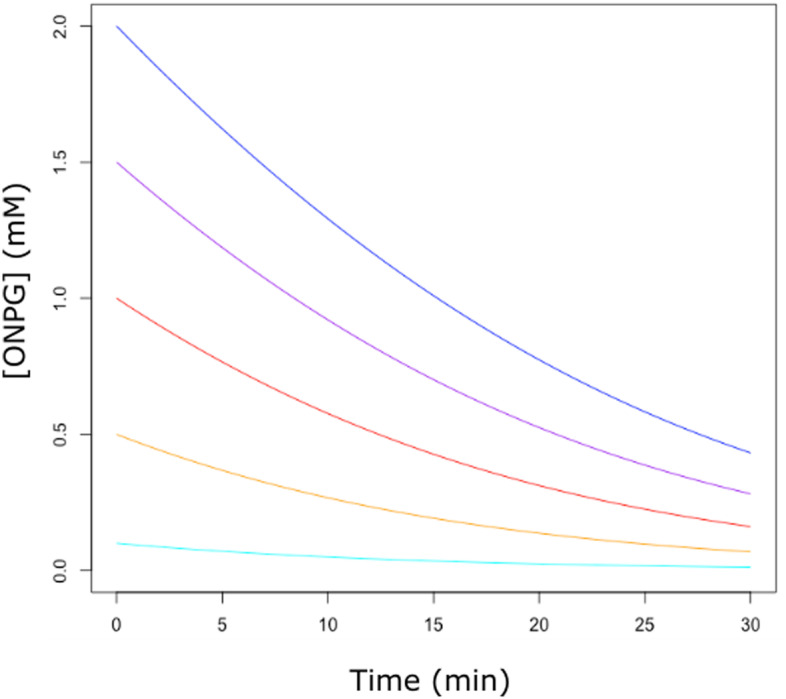
Progress curves for the *β*-galatosidase catalyzed hydrolysis of ONPG. Each curve has been generated by the command *sE.progress* (So, time = 30, *Km* = 2.5, *Vm* = 0.18) where S_o_ represents the initial concentration of ONPG (2 mM: blue curve; 1.5 mM: purple curve; 1 mM: red curve; 0.5 mM: orange curve and 0.1 mM: cyan curve)

Unfortunately, Eq. (2) is a so-called implicit equation, which cannot be solved analytically for [*S*] = *f(t)* with elementary mathematics [23]. However, implicit equations where the variable appears accompanied by the natural logarithm of itself, can be solved resorting to the mathematical concept of the Lambert’s W function, which in the case of the integrated Michaelis–Menten equation will lead to the so-called Schnell-Mendoza equation (see the vignette “Michaelis–Menten and the Lambert W function” for details) [22, 24]. Despite the advantages of this approach, the reluctance of many biologists to use non-elementary mathematics and the lack of free and easy-to-use software that implements such an approach, have made it to go unnoticed outside of the specialist community. To help reverse this situation, the package renz includes two functions based on the above exposed approach. On one hand, the function *fE.progress()* takes as input the experimental ($t, \left[ S \right]$) data and fit them to an explicit solution of the integrated Michaelis–Menten equation, providing, in this way, an unbiased estimation of K_M_ and V_max_. On the other hand, the function *sE.progress()* takes as input the K_M_ and V_max_ values for a Michaelis–Menten enzyme and simulate the substrate progression curve (the user is given the option to choose the type and magnitude, including null, of the experimental error). This second function may be useful in the context of enzymology teaching (Fig. 5).

## Conclusions

While several sophisticated programs that provide support to practicing enzymologists exist, most researchers outside the enzymology field resort to general purpose regression and graphing software to analyze enzyme kinetic data. However, this practice, which often requires prior manipulation (transformation) of the original data to be analyzed, is time-consuming and prone to human error. Even worse, the estimated kinetic parameters can be highly biased when experimental error is not properly accounted for, which is often the situation when nonspecialized software is used. Thus, to facilitate a rigorous but still easy-to-implement analysis of enzyme kinetic data, while avoiding common pitfalls, we have developed a cross-platform software package, renz, that is distributed as free and open-source code. Besides to automate the classical linearization methods (Lineweaver–Burk, Eadie-Hofstee, Hanes-Woolf and Eisenthal-Cornish-Bowden), features include the ability to carry out weighted regression analyses that, in most cases, substantially improves the estimation of kinetic parameters. To avoid data transformations and the potential biases introduced by them, the package also offers functions that directly fit data to the Michaelis–Menten equation, either using ([*S*], *v*) or (*t*, [*S*]) data (differential and integrated forms of the Michaelis–Menten equation, respectively). Utilities to simulate the substrate concentration evolution over time, making use of the Lambert W function, are also provided. The package is accompanied by five vignettes, which are intended to guide the user in choosing the appropriate method in each case, as well as providing basic theoretical notions to allow a critical interpretation of the obtained results and their robustness. Overall, the current software, in addition to serving as a convenient program for a broad spectrum of researchers in the life sciences field, is also a useful resource for teaching enzymology.

## Availability and requirements

Project name: R package for the analysis of kinetic data from enzyme-catalyzed reactions (renz).

Project home page: https://bitbucket.org/jcaledo/renz.

Operating system(s): Platform independent.

Programming language: R.

Other requirements: N/A.

License: GPL-2, GPL-3.

Any restriction to use by non-academics: N/A.

## Data Availability

The program described herein is freely available at https://cran.r-project.org/web/packages/renz. In addition, all source code is present in the associated Bitbucket repository, located at https://bitbucket.org/jcaledo/renz. The kinetic data use for the case-study are included in the package and can be obtained as a data frame typing ‘ONPG’ into the R console.

## Abbreviation

ONPG: o-Nitrophenyl-β-d-galactopyranoside
